# Seroprevalence rate of Poliovirus antibodies among the Healthy and Protein Energy Malnutrition children

**DOI:** 10.12669/pjms.312.5366

**Published:** 2015

**Authors:** Aliya Yousuf, Skindar Ali Syed Shah, Imtiaz Ahmed Syed Jaffery, Syed Azher Ahmed, M.A Basit Khan, Mohammad Aslam

**Affiliations:** 1 Dr. Aliya Nemat, MPH, Baqai Institute of Health Science, Baqai Medical University, Karachi, Pakistan; 2 Syed Skindar Ali Shah, Msc Medical Technology, Shahdadpur Institute of Medical Science, Sind, Pakistan; 3 Dr. Syed Imtiaz Ahmed Jaffery, MPH, Baqai Institute of Health Science, Baqai Medical University, Karachi, Pakistan; 4 Dr. Syed Azher Ahmed, PhD (Pathology), Post graduate Program Baqai Medical University, Karachi, Pakistan; 5 Dr. M.A. Basit Khan, MPH, Baqai Institute of Health Science, Baqai Medical University, Karachi, Pakistan; 6 Dr. Mohammad Aslam, FCPS (Community Medicine), Baqai Institute of Health Science, Baqai Medical University, Karachi, Pakistan

**Keywords:** Malnutrition, Protein energy malnutrition, IGg antibodies, Polio

## Abstract

**Objective:**

To study the association between Protein energy malnutrition and polio-specific immunoglobulin G antibodies production among children in Gadap Town Karachi, Pakistan.

**Methods:**

Comparative cross sectional survey conducted at fixed EPI center and Pediatric OPD of a tertiary care hospital Karachi. Children were selected by convenient sampling method during the period from 17 March to 17 May 2013. It was ensured that they must have received more than seven oral polio vaccine doses as eligibility criteria for the study. A total of 170 blood samples were collected and tested for the presence of polio-specific IgG antibodies using Poliomyelitis IgG ELISA Test Kit produced.

**Results:**

Statistically significant relation was found between PEM and IgG antibodies production OR (P = 0.000). Overall Seroprevalence rate among the study population was 98.8%, PEM group 97.6% and healthy group 100%.

**Conclusion:**

The study demonstrated that there is a need to focus on the protein energy malnutrition among the children as an immunization strategy for the 100% seroprevalence rate in all population against polio in Pakistan.

## INTRODUCTION

Disease eradication is long term sustainable management as compared to disease control and elimination program. After successful small pox eradication, poliomyelitis is choice for next eradication disease. While after Global polio eradication initiative program since 1988 mortality and morbidity due to polio has reduced, complete eradication of the polio would save more than forty billions of dollars globally. This income could be used for other public health program. This will not only be good for poor countries but also give positive effect on public health program.^1^

Globally polio is almost eradicated except from three countries Pakistan, Nigeria and Afghanistan, India eradicated the polio in 2012.^2^ According to WHO independent monitoring report 2013: Pakistan is hardest country regarding polio eradication, in 2013 Pakistan had highest prevalence of polio and Gadap town Karachi was labeled as high risk area regarding polio. Globally Malnutrition is the major cause for failure of all vaccination.^3^ According to Independent Monitoring Board of the Global Polio Eradication Initiative, Tenth Report 2014 on 28th of October 2014, globally 257 polio cases were reported in three polio endemic countries, of which 220, wild polio cases were reported from Pakistan.

According to “Jack Dean”, presently 165 million children are universally, chronically malnourished. This preventable state has affected one in every four children. Malnutrition cause 2.3 million children deaths per year, an average of one death per every 15 seconds. According to WHO majority of expected malnourished children live in developing countries. Less than 30% of them are under the age of five years and half of them are suffering from PEM.^4^

Research studies from Aga Khan University, UNICEF and National Nutritional Survey of Pakistan, 2011, Islamabad shows that Malnutrition has been known as the main difficulty for polio eradication among under five years old children in Sind Province.^5^

In its 41^st^ meeting in 1988, World Health Assembly passed a declaration according to which “OPV is exclusively used worldwide for polio eradication for some reasons. It is cheaper than Intramuscular polio Vaccine (IPV) and can be easily orally administered”.^6^ However, worldwide, the oral vaccine doesn’t effectively develop immunity against polio, particularly in malnourished children.^7^

Some data from WHO has also highlighted that malnutrition hinders the battle against polio because OPV produces four times less immunity in malnourished children as compared to well-nourished. Since 40% of under-five children are malnourished in Pakistan, it is very difficult to eradicate polio from Pakistan.^9^

Therefore, our priority should be focused on preventing malnutrition in this group of population.^5^ In this context, malnutrition seems to be the main problem in polio eradication in under-five children in Sind Province.^10^

## METHODS

### Ethical Consideration

Ethical clearance for the study was taken from the Baqai Ethical Research Review Committee (BERRC) and also from the Baqai Ethical Committee. A written permission was also taken from the Medical Superintendent of the Fatima Hospital BMU and the head of the pediatric department of the Fatima Hospital BMU. Informed Consent was taken from the parents and guardians of all selected children before the questionnaire was filled in and before the blood samples were collected.

### Survey procedure and Laboratory Analysis

A written informed consent was first obtained from all parents and guardians before inclusion of their children in the study. Next, demographic data and other relevant information of each participant were obtained through a questionnaire. Then, about 4 ml of blood was aseptically drawn by venipuncture after swabbing the area of interest for sample collection with alcohol. The serum was then separated from the blood by allowing clotting and centrifuging. Finally, the serum samples were labeled and stored at -20ºC until processed. The polio IgG antibody ELISA test kit, manufactured and described by DEMEDITEC Diagnostic GmbH Germany, was used for the detection of specific IgG antibodies against polio in the serum of children with strict adherence to the manufacturer’s instruction manual.

### Study setting

The study was conducted at EPI center at the Baqai Medical University and the Pediatric Outpatient Department (OPD) of Fatima Hospital at Baqai Medical University (BMU). The Hospital was selected because it is the only teaching and welfare hospital for this rural community, Gadap Town, Karachi. It provides health services to the population of eight union councils of the Town, Karachi. The blood samples were collected at the Fatima Hospital Laboratory, Baqai Medical University and analyzed at the Baqai Hospital Lab, Nazimabad, Karachi.

### Study Population

Children between the age of 10 months to 59 months who either attended the pediatric Outpatient Department (OPD) with some mild illness, or attended EPI (expanded program for immunization) fixed center at Fatima Hospital BMU for vaccination. These subjects of the study were recruited from 17th of March, 2013 to 17th of May 2013 as the study population. It was ensured that they must have received more than seven oral polio vaccine doses and fulfill the eligibility criteria of study. A total of 170 blood samples were collected.

### Sample size

The sample size was calculated by using sample size determination in health studies by WHO with 95% confidence interval and 5% level of significance. The proportions of antibodies production among malnourished subjects [“Evaluation of the response to vaccination against poliomyelitis and measles in malnourished children in Morocco”^120^] was 59.5% in malnutrition children and 97.1% in well-nourished children. The sample size was calculated to be 40 subjects in each group. In such a small sample size, the chance of type 1 and type 2 errors are increased. To reduce these errors in the study, we increased the sample size to 85 subjects in each group.

### Data collection

#### Research tools

1. Poliomyelitis Virus IgG Elisa Kit (enzyme-linked immunosorbent assay) was used for elicitation of a quantitative analysis of Polio-specific Immunoglobulin G antibody in the serum of target population. The DEMEDITEC Polio IgG Antibody ELISA (enzyme-linked immunosorbent assay) was the standard test kit for the analysis of level of Polio-specific Immunoglobulin G antibody in the serum of target population.
  According to the instruction manual of this kit the following controls were applicable:
  - **Negative Control.** The serum contained no IgG antibodies against polio (1 to <10 IU/ml)
  - **Cut-Off Standard.** The serum contained a low concentration of IgG antibodies against polio (10 to <50IU/ml)
  - **Weak Positive Control.** The serum contained a medium concentration of IgG antibodies against polio (50 to <150 IU/ml)
  - **Positive Control.** The serum contains a high concentration of IgG antibodies against polio (>150 IU/ml)
2. The questionnaire of the study.
3. Gomez method for evaluation of the nutritional status of the children.

### Inclusion criteria

Healthy children from the Fixed EPI center at Baqai Medical University. Mildly ill children were from the pediatric OPD of the Fatima Hospital, Baqai Medical University. They were found with or without Protein Energy Malnutrition according to Gomez classification between the age of 10 months and 59 months who were selected. Children were resident of Gadap town and, who had received more than seven doses of Oral polio Vaccine in polio vaccination before the start of this study. Children, whose vaccination dates were recorded in the clinic records, immunization cards, or birth certificates and certified either by the staff of the Fixed EPI Center, Fatima Hospital, or by a lady health worker of the Basic Health Unit, Sind Government, or by the guardians of the child (mother, father, grandfather, grandmother, uncle, aunt, sister or brother). Children, their parents or guardians gave informed consent.

## RESULTS

The clinical and laboratory data was analyzed through statistical package SPSS version 19. The data is presented as mean, median, mode, frequencies Seroprevalence rate, cross tabulation, chi-square and P- value in table and figures.

Total 170 samples were tested for polio-specific IgG antibodies in which 168(98.8%) samples were positive and two subjects were seronegative. These two subjects belong from PEM grad III group as shown in Fig.I: An overall seroprevalence rate of 98.8% among the study population was noted. Of which, PEM group seroprevalence rate was 97.6% and the healthy group had a seroprevalence rate of 100%.

**Fig.I:**
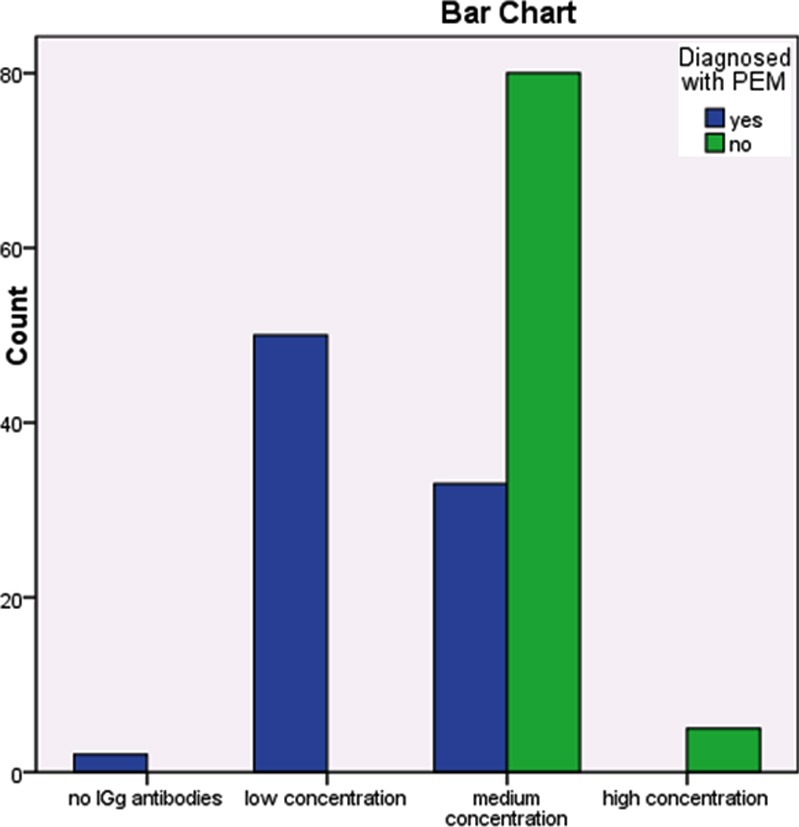
Level of IGg antibodies against polio.

Fig.II shows seroprevalence rate among the PEM groups, 97.6%, while in three grads of PEM groups, grade-I had100%, PEM grade-II had 100% and PEM-III had 95%. Statistically significant relation was found between PEM and IgG antibodies production OR (P = 0.000), because IgG antibodies production was decreased in high grads of PEM (PEM grad-II and grad-III).

**Fig.II:**
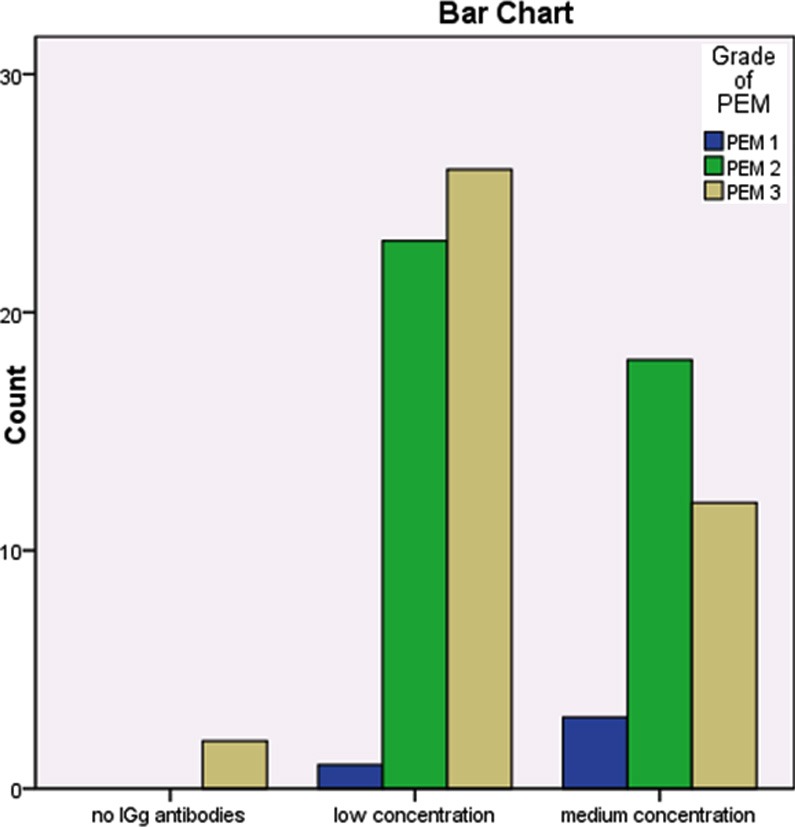
Level of IGg antibodies against polio.

### Interpretation

Table-I shows: mean of age variable was 32.34months, mode of age variable was 48, median 30 and std. deviation of age variable was 14.47 of total 170 study children ages.

**Table-I:** Interpretation of mean, median, mode, std. deviation and frequencies distributions.

Statistics		
Age of study children		
N	Valid	170
	Missing	0
	Mean	32.28
	Median	30.0000
	Mode	48.00
	Std. Deviation	14.83
	Minimum	10.00
	Maximum	58.00

## DISCUSSION

A total of 170 serum samples were collected and analyzed in this study for polio-specific IgG antibodies, with a Seroprevalence of 98.8% with medium concentration of IgG antibodies levels. Similar serologic surveys had been carried out in the Aga khan university Karachi Pakistan^10^, south-western Nigeria^11^ and Jos University Teaching Hospital Nigeria.^12^

The high seroprevalence of 98.8% recorded in this study indicates children have effectively responded to the vaccine being used in the ongoing polio eradication initiative. These findings are similar with United States study in which it was reported that greater than 90% of school age children, adolescent and young adults had detectable antibodies to Poliovirus.^13^

However, the percentage is higher than the findings in Ibadan, Nigeria, in which only 59.6% herd immunity for the three Poliovirus serotypes were recorded.^12^ and, that from the time of their study in 1990 to this time, immunization coverage have increased to produce a higher seroprevalence. The level of awareness on the need to get children immunized against the virus has now day by day increased, as in this study; almost all respondent know about polio and OPV campaign, the antibodies detection rate are to some extent lesser than earlier study conducted in Egypt.^14^

Seropositivity among PEM group 97.6% and healthy group 100%, no variation had been seen among this study finding and other study finding like in MA Habib and Sofia study in Pakistan^10^, Egypt^14^, and H. Caidi, F. Bennis study in Marcoo, Seroprevalence among healthy group 94.1% and among PEM group 37.8% and PEM-III had 21.4% seroprotection rate.^2^

### Limitation of study

This was a comparative survey conducted in community of all higher risk and polio free Union Councils of Gadap town. Due to security concern it was not possible to conduct this study in community by door to door survey. Neither in, all fixed EPI centers of Gadap town, primary health care units and basic health units of Gadap town. Under such circumstances blood sampling of child and interview of respondents was not possible. There was threat from Pakistan Taliban leader of these areas. They gave the fatwa against polio campaign, saying that polio vaccination is against the will of God and it is American lobby to reduce the Muslim population. During the last one year many members of polio eradication team were killed especially in Gadap town Karachi Pakistan.^15^ Study site was chosen on availability of security. As such may be that children or study population which we selected for sample size did not representative all children of the same age in this age and same diagnosis with PEM. Children who received care at OPD and EPI center may have higher immunization coverage and seropositivity than children who received care of other EPI centers of Gadap town. There was selection bias. Children who took more than seven OPV doses in all polio eradication campaigns at the time of study were selected randomly. There was chance of recall bias. Samples were analyzed at the Baqai Hospital Lab Nazimabad Karachi. It is ISO certified lab in Pakistan. As already mentioned this study was conducted without any collaboration as compared with other study that was conducted in AKU Pakistan and Egypt. Those studies blood samples were analyzed in Centre for Disease Control laboratory (CDC) in Atlanta, but result of this study are almost the same as the above mentioned study.

## RECOMMENDATIONS

- Screening of all types of malnutrition and proper identification, prevalence of children of with malnutrition.
- Management and treatment of Malnutrition at primary health care services.
- There is need to improve knowledge, awareness and practice regarding malnutrition of Primary health care unit, basic health care unit personnel as well as community specially mother.
- The antibodies production rate was low in PEM group, as such there is need to introduce some other Interventions in polio eradication program for this group.
- PEM group gave poor response to OPV hence there is need to give this group IPV (injectable polio vaccine) along with OPV and different micronutrition deficiencies like Zinic, iron.
